# Comprehensive analysis reveals MCM4 as a biomarker for guiding therapies and immunomodulatory role in skin cutaneous melanoma

**DOI:** 10.7150/jca.117471

**Published:** 2025-08-11

**Authors:** Wei Ou, Jiang Zhou, Zhenhua Huang, XinLiang Yang, Xiaohong Liu, Wenjian Zuo, ZhiYong Luo, Min Su

**Affiliations:** 1 Department of Biochemistry and Molecular Biology, School of Life Sciences, Central South University, Changsha 410008, China.; 2 Department of Pharmacy, Yueyang Central Hospital, Yueyang 414000, China.; 3 Hunan Key Laboratory of Cancer Metabolism, The Affiliated Cancer Hospital of Xiangya School of Medicine, Central South University/Hunan Cancer Hospital, Changsha 410013 Hunan, China.

**Keywords:** Skin cutaneous melanoma, MCM4, Prognosis, Immunotherapy, Immune infiltration, Drug sensitivity

## Abstract

**Background**: Skin cutaneous melanoma (SKCM) is a malignant tumor characterized by aggressive invasion and a high tendency for metastasis. This study explores the potential of MCM4 as a biomarker for SKCM and its impact on the tumor microenvironment (TME).

**Method**: A comprehensive analysis of MCM4 was conducted using public databases to characterize the expression, genomic alterations, and clinical significance of MCM4 in pan-cancer, including SKCM. Bioinformatics tools were employed to identify upstream regulators of MCM4. The functional mechanisms of MCM4 in SKCM were explored through correlation, differential, and enrichment analyses. Immune infiltration and drug sensitivity were assessed to understand the role of MCM4 in the TME and its potential therapeutic implications. Functional experiments were performed in A375 and SK-MEL-28 cells.

**Results**: MCM4 were significantly upregulated in tumors. Survival curves indicated that patients with high MCM4 expression had poor survival advantage. SRF was identified as a potential transcription factor regulating MCM4. Functional enrichment revealed a positive correlation between MCM4 and cell cycle-related pathways, and a negative correlation with immune effector process-related pathways. High MCM4 expression was associated with "cold" tumor characteristics. Immunotherapy response analysis demonstrated higher response rates in patients with low MCM4 expression. Drug sensitivity analysis suggested potential therapeutic drug options based on MCM4 expression. Functional experiments confirmed the oncogenesis effects of MCM4 in SKCM cells.

**Conclusion**: MCM4 is a potential prognostic biomarker and predictor of immunotherapy response in SKCM patients.

## Introduction

Skin cutaneous melanoma (SKCM) is a particularly aggressive malignancy, characterized by its high metastatic potential and poor prognosis in advanced stages 1. Although SKCM accounts for only 1% of skin cancer cases, it is responsible for the majority of skin cancer-related deaths. Patients with advanced or metastatic melanoma have a dismal outcome, with a 5-year survival rate below 25% 2. Recent advances in targeted therapies, including BRAF and MEK inhibitors, and immunotherapies such as anti-CTLA-4 and anti-PD-1 agents, have improved patient outcomes. However, challenges persist due to drug resistance and disease recurrence 3-5. Therefore, gaining a deeper understanding of the molecular mechanisms of SKCM and identifying reliable biomarkers are crucial for enhancing prognostic assessment and personalized treatment strategies.

Genomic amplification is a hallmark of cancer and accumulates during tumor progression. To maintain genome replication and stability, tumor cells rely on a large number of chromosomal stability-related proteins 6. MCM4, a key component of the pre-replication complex, is indispensable for initiating DNA replication. Among the MCM protein family, MCM4 is considered the most conserved protein throughout evolution 7. By unwinding DNA double strands and facilitating replication fork progression, MCM4 plays a pivotal role in regulating DNA replication and maintaining genome stability. Increased expression of MCM4 has been observed in various tumors, where it serves as a reliable diagnostic and prognostic biomarker 8-11. These findings make MCM4 an attractive target in cancer research. It is worth noting that an immunohistochemical analysis has confirmed the high expression of MCM4 in superficial spreading melanoma (SSM) 12. However, since SSM is just one subtype of SKCM, further research is still needed to investigate the expression of MCM4 in other types of SKCM, as well as the prognostic value, function, and molecular mechanisms of MCM4 in SKCM.

This study aims to comprehensively analyze the expression levels, genomic alterations, and clinical significance of MCM4 in SKCM. By leveraging extensive datasets from The Cancer Genome Atlas (TCGA), Gene Expression Omnibus (GEO), and the DepMap Portal, we seek to delineate the relationship between MCM4 expression and SKCM progression, treatment response, and overall patient outcomes. Furthermore, we investigate the potential of MCM4 to serve as a predictor for immunotherapy efficacy, considering the increasing role of immunomodulatory treatments in SKCM management.

## Materials and Methods

### Expression and genomic alteration analyses of MCM4

We obtained TCGA-SKCM tumor samples and controls from the UCSC Xena portal 13, comprising 461 tumor samples from the TCGA and 557 normal skin samples from GTEx. Additionally, data from GSE46517, comprising 73 metastatic melanomas, 31 primary melanomas, and 17 normal controls, and GSE98394, which included 51 primary melanomas and 27 normal controls, were sourced from the GEO database (https://www.ncbi.nlm.nih.gov/geo/). The demographic information of the patients was summarized in Supplement Table 1. Pan-cancer comparisons of MCM4 mRNA expression levels were conducted using the TIMER 2.0 tool 14, while protein expression levels of MCM4 across pan-cancer were analyzed using the UALCAN platform 15. The genomic landscape of MCM4, including mutations and copy number variations, was explored through the cBioPortal 16, where the relationship between mutation sites and protein structure was also examined.

### Prognostic analysis of MCM4

We conducted survival analyses using Kaplan-Meier (KM) curves to evaluate MCM4's prognostic significance. The BEST database 17 was employed to analyze the survival effects of MCM4 on SKCM across multiple datasets. Patients were categorized into high and low MCM4 expression groups based on an optimal threshold, and the significance of survival differences was assessed using the log-rank test. Additionally, the Sangerbox 3.0 tool 18 was utilized to investigate the relationship between MCM4 expression and overall survival (OS) in pan-cancer patients. Univariate Cox regression analysis was performed to calculate the hazard ratio (HR) and 95% confidence intervals (CI).

### Identification of potential transcription factors (TFs)

To identify upstream regulators of MCM4 expression, we utilized several databases, including hTFtarget 19, Cistrome 20, JASPAR 21, and ENCODE 22, to predict potential TFs. Differential expression analysis was performed to further screen candidate TFs. Spearman correlation analysis was then applied to assess the relationship between MCM4 and the identified TFs across pan-cancer datasets.

### Functional analysis of MCM4 in SKCM

Spearman correlation analysis was performed to identify the top 50 genes most positively and negatively correlated with MCM4 expression. The top 500 genes co-expressed with MCM4 were subjected to Gene Ontology (GO) annotation (https://geneontology.org/). Additionally, SKCM patients were categorized into high-expression and low-expression groups based on the median expression level of MCM4. The Gene Set Enrichment Analysis (GSEA) algorithm 23 was used to analyze enrichment scores for terms related to the cell cycle and immune processes.

### MCM4 and immune infiltration analysis

We evaluated the impact of MCM4 expression on immune infiltration in the SKCM tumor microenvironment (TME) using the ESTIMATE algorithm for immune and stromal scores. The CAMOIP database 24, employing the MCPcounter algorithm, was conducted to quantify immune and stromal cell fractions in TCGA-SKCM.

### Potential drug screening

PRISM 25 is a pharmacogenomic database that includes data from over 500 cell lines and more than 4,500 drugs. Using PRISM data alongside SKCM transcriptomes, we applied the ridge regression algorithm to predict the susceptibilities to small molecule drugs. Spearman correlation analysis was then conducted to evaluate the relationship between MCM4 expression and the predicted half-maximal inhibitory concentration (IC50).

### Cell culture

The SK-MEL-28 and A375 human SKCM cells, sourced from the Cell Bank of the Chinese Academy of Sciences (Shanghai, China), were cultured in Dulbecco's modified Eagle medium (DMEM) supplemented with 10% fetal bovine serum at 37 °C in a 5% CO2 humidified atmosphere.

### Knockdown of MCM4 by sgRNA

Two sgRNA sequences targeting MCM4 were designed, and the sgRNA oligos were phosphorylated and ligated. The lentiviral CRISPR V2 plasmid was dephosphorylated and digested, and the resulting fragments were separated by agarose gel electrophoresis. The larger fragments were then excised and recovered from the gel. The phosphorylated sgRNAs and the recovered V2 plasmid fragments were mixed and ligated according to the manufacturer's instructions. The ligation products were transformed into Stbl3 competent cells, and single colonies were selected for plasmid extraction and sequencing to confirm successful cloning. The sequences of the sgRNAs targeting human MCM4 are as follows: sgRNA-1, 5′-CCGATCATTCTTCTCTGACAA-3′; and sgRNA-2, 5′-GCGGTGCTAAAGGACTACATT-3′.

### Reverse transcription quantitative polymerase chain reaction (RT-qPCR) and western blot (WB)

The knockdown efficiency was assessed using RT-qPCR and WB, following our previously established protocol 26, 27. Briefly, protein extraction was performed using ultrasonication, followed by the addition of 2× protein loading buffer and denaturation at 100°C. Proteins were separated via electrophoresis and transferred onto an NC membrane. The membrane was blocked and incubated sequentially with primary and secondary antibodies. Detection was performed using an imaging system, and quantitative analysis was carried out using ImageJ software.

### Cell counting kit-8 (CCK8) assay

Following transfection with either control or MCM4 knockdown plasmids, SK-MEL-28 and A375 cells were plated in 96-well plates at a density of 2,000 cells per well and cultured for 24 hours. Subsequently, CCK-8 reagent was added. After 2 hours of incubation, absorbance at 450 nm was measured to assess cell viability.

### Plate colony formation assay

SK-MEL-28 and A375 cells were seeded in 6-well plates at a density of 500 cells per well and cultured for approximately 14 days. The colonies were then fixed with 4% paraformaldehyde for 15 minutes and stained with 0.5% crystal violet (Sigma-Aldrich, Shanghai, China) for 20 minutes. Colony numbers were quantified by measuring absorbance at 450 nm using spectrophotometry.

### Flow cytometry assay

A375 and SK-MEL-28 cells were infected with lentiviral shRNAs for 48 hours. Following infection, 2 × 10^5^ cells were harvested by centrifugation and resuspended in 500 µL of 1× Annexin V Binding Buffer. Subsequently, 5 µL of Annexin V-FITC and 5 µL of Propidium Iodide were added. The cells were incubated in the dark at room temperature for 5 minutes and analyzed using a BD FACSVerse^TM^ flow cytometer (Franklin Lakes, NJ, USA).

### Statistical analysis

Statistical analyses were carried out using software such as R and GraphPad Prism. Differences between groups were evaluated using two-side Student's t-test or one-way ANOVA, as appropriate, with survival outcomes analyzed via Kaplan-Meier curves and log-rank tests. The significance level was set at p < 0.05.

## Results

### Expression analysis of MCM4

**Figure 1:**
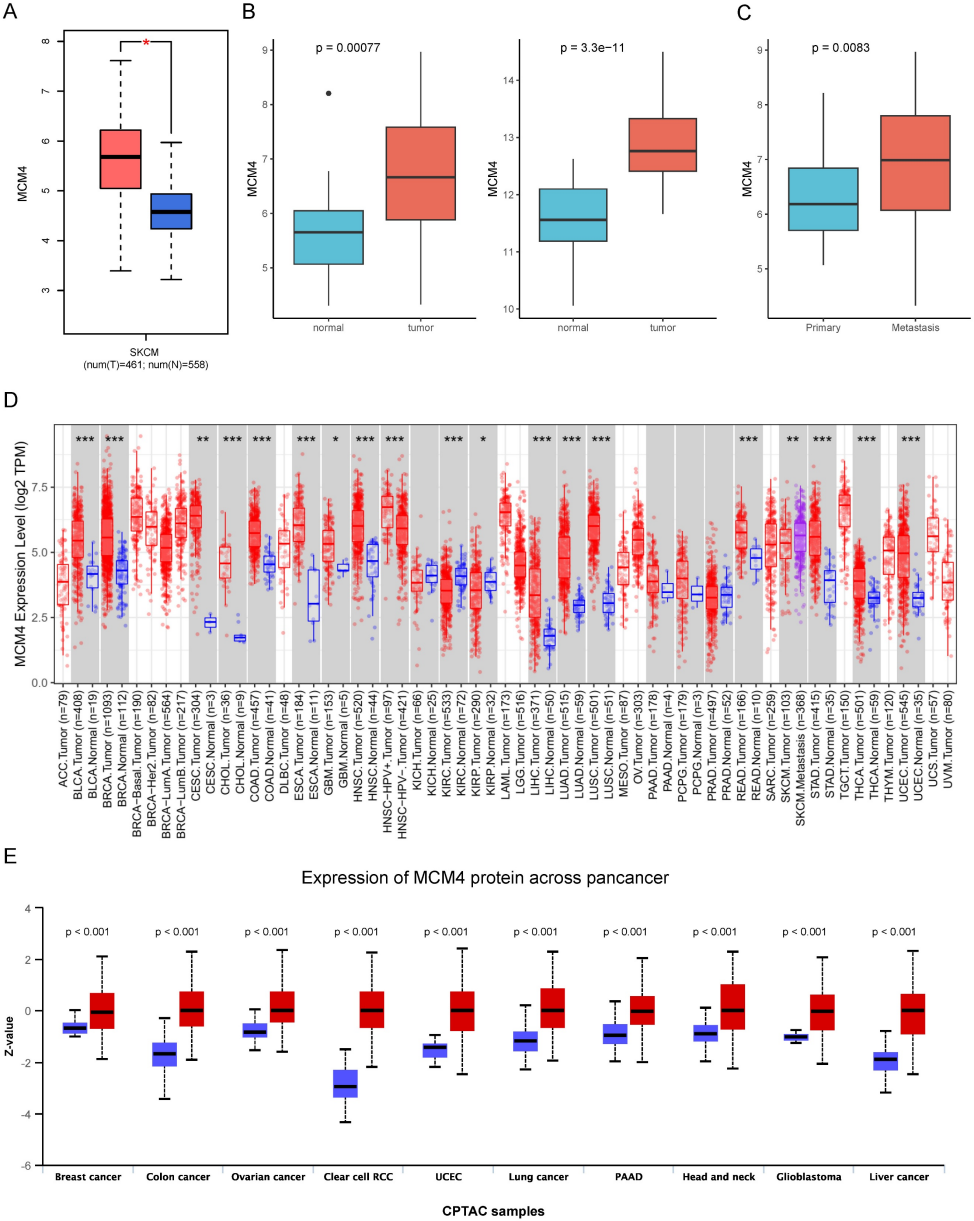
** Analysis of MCM4 expression in SKCM and pan-cancer.** (A) The expression levels of MCM4 in SKCM compared to controls. (B) Independent validation of MCM4 expression in SKCM using GSE46517 and GSE98394 datasets, respectively. (C) The MCM4 expression was elevated in metastatic SKCM. (D) Boxplot of MCM4 expression across pan-cancer. (E) Comparison of MCM4 protein levels across pan-cancer in CPTAC database. *p < 0.05, **p < 0.01, ***p < 0.001.

We first investigated the expression of MCM4 in SKCM by integrating data from the TCGA and GTEx databases. Our analysis revealed that MCM4 expression was significantly elevated in tumor tissues (Figure 1A). This finding was independently validated using two distinct datasets (GSE46517 and GSE98394) (Figure 1B). Furthermore, we observed an even greater increase in MCM4 expression in metastatic SKCM samples (Figure 1C). Pan-cancer analysis addresses the limitations of studying individual cancer types in isolation, providing insights with broader implications across multiple cancer types 28. We conducted pan-cancer expression analysis and found that MCM4 expression was markedly elevated in most cancer types (Figure 1D). In the TCGA-SKCM cohort, metastatic tumors exhibited higher MCM4 expression compared to primary tumors. We further compared the protein levels of MCM4 and the results demonstrated the upregulation of MCM4 (Figure 1E).

### Genomic alteration of MCM4 and stemness analysis in pan-cancer

The genetic changes of oncogenes are closely associated with cancer development and progression (24). Therefore, we inquired about various changes in MCM4 in the cBioPortal database, including mutations, structural variations, amplifications, deep deletions, and multiple variations (Figure S1A). The overall alteration frequency of MCM4 was 4.0%, with the highest occurrence observed in uterine carcinosarcoma (~16%). We further explored the relationship between mutation sites and the MCM4 protein structure, identifying the T629Qfs*18 mutation in the MCM domain as the most prevalent (Figure S1B).

Tumor stemness is an indicator of cancer stem cell-like characteristics that reflects the degree of oncogenic dedifferentiation 29. To assess the impact of MCM4 on tumor stemness across pan-cancer, we conducted a Spearman correlation analysis, revealing an extensive positive correlation between MCM4 expression and stemness (Figure S1C). In melanoma, the correlation coefficient was 0.318, with a p-value < 0.01, suggesting that MCM4 may contribute to the stemness characteristics of SKCM.

### MCM4 was a risk factor in SKCM

**Figure 2:**
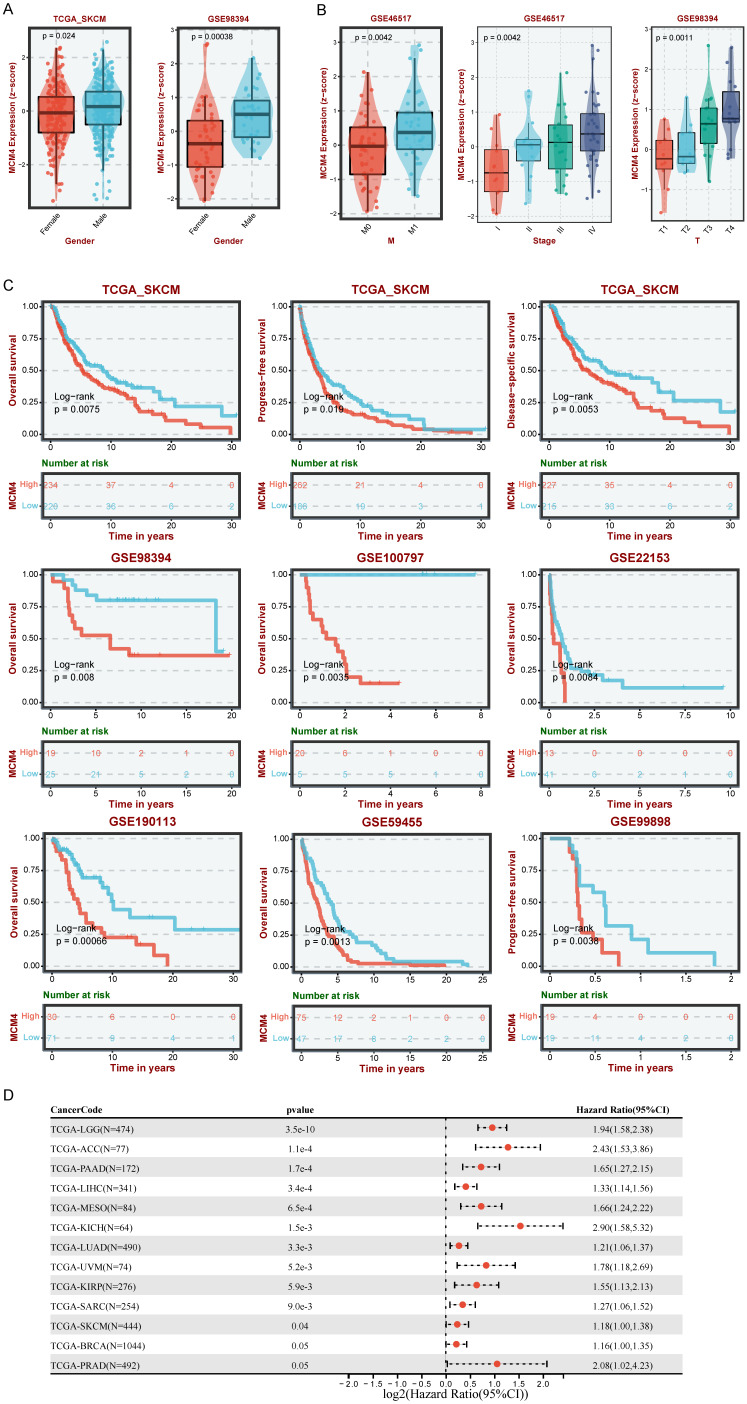
** Analysis of MCM4's clinical implications in SKCM.** (A) The comparison of MCM4 levels between male and female SKCM patients. (B) Association between MCM4 expression and T, M, and stage in SKCM. (C) Kaplan-Meier (KM) curves highlighting that elevated MCM4 expression indicating shorter survival outcomes in SKCM. (D) Forest plot of univariate Cox regression results in pan-cancer.

To assess the clinical relevance of MCM4 in SKCM, we examined its expression in relation to various clinical characteristics. Boxplots revealed that MCM4 expression was higher in males compared to females (Figure 2A). Additionally, MCM4 expression significantly increased with tumor progression in both the GSE46517 and GSE98394 datasets (Figure 2B). We further investigated the clinical impact of MCM4 expression on patient outcomes by analyzing data from seven melanoma cohorts in the TCGA-SKCM and GEO databases. KM curves indicated that patients with high MCM4 expression had a poor survival advantage (Figure 2C). To evaluate the prognostic significance of MCM4 across pan-cancer, we performed univariate Cox regression analysis. The forest plot showed that MCM4 was a risk factor in 11 types of tumors (Figure 2D). These findings highlight MCM4 as a promising prognostic biomarker for SKCM.

### SRF as a potential regulator of MCM4 in SKCM

**Figure 3:**
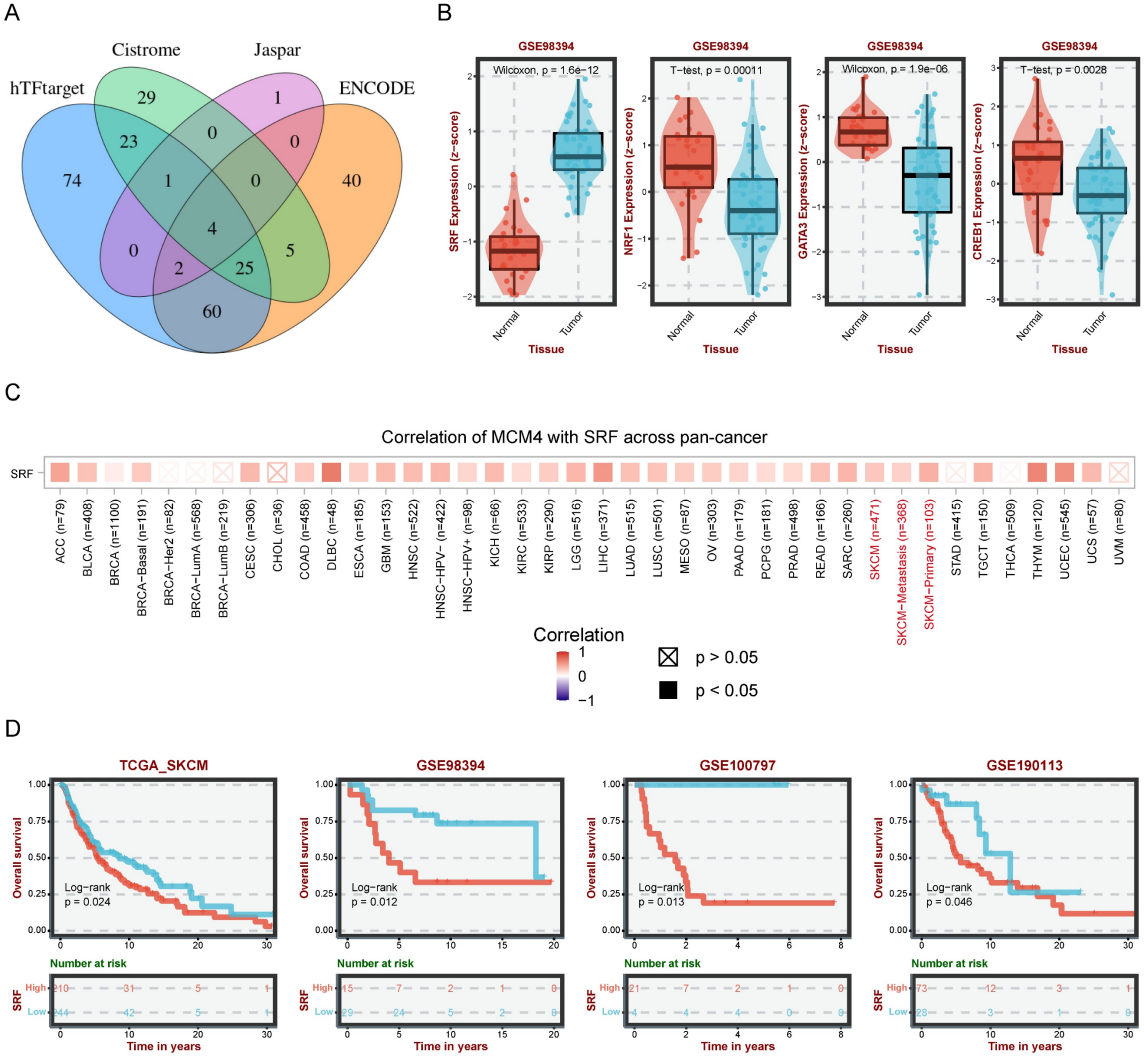
** Prediction of transcription factors (TFs) regulating MCM4.** (A) Venn diagram illustrating the overlap of potential TFs from four databases (hTFtarget, Cistrome, JASPAR, and ENCODE). (B) The expression levels of four TFs in SKCM. (C) Pan-cancer correlation analysis between MCM4 and SRF. (D) Survival analysis revealing poor outcomes in patients with high SRF.

To uncover the upstream regulatory mechanisms of MCM4 upregulation in SKCM, we performed a comprehensive analysis using four web tools (hTFtarget, Cistrome, Jaspar, and ENCODE) and identified 4 candidates (SRF, NRF1, GATA3, and CREB1) (Figure 3A). Differential analysis showed significantly elevated SRF in SKCM, while the other three TFs were downregulated (Figure 3B). These findings prompted us to focus on SRF for further investigation. Pan-cancer analysis revealed a significant positive association between SRF and MCM4 (Figure 3C). Moreover, KM analysis indicated that elevated SRF expression was associated with worse outcomes in SKCM patients (Figure 3D).

**Figure 4:**
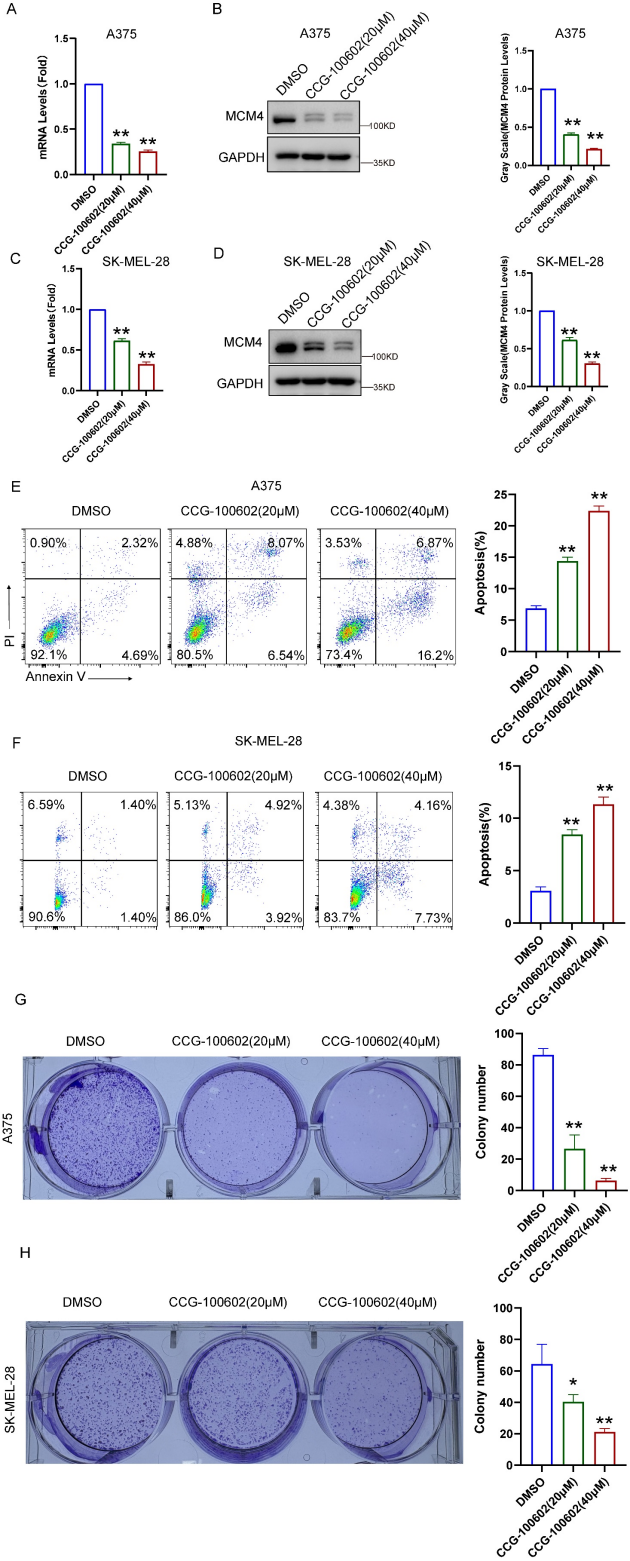
** CCG-100602 inhibits MCM4 expression and SKCM cell proliferation.** (A, C) The qPCR results of MCM4 mRNA levels in A375 (A) and SK-MEL-28 cells (C). (B, D) Western blot (WB) analysis of MCM4 protein expression in A375 (B) and SK-MEL-28 cells (D). (E, F) Flow cytometry detection of cell apoptosis in transfected SKCM cells. (G, H) Colony formation assay comparing colony numbers of SKCM cells with or without CCG-100602 treatment.

To validate these bioinformatics findings, we conducted experiments using the SRF inhibitor CCG-100602. In A375 cells, qPCR and WB assays demonstrated a dose-dependent decrease in both MCM4 mRNA and protein levels following CCG-100602 treatment (Figure 4A, B). This result was replicated in SK-MEL-28 cells (Figure 4C, D). Furthermore, CCG-100602 induced apoptosis and inhibited cell proliferation in a dose-dependent manner (Figure 4E-H). These results suggest that SRF plays a role in regulating MCM4 expression in melanoma.

### Functional analysis of MCM4 in SKCM

**Figure 5:**
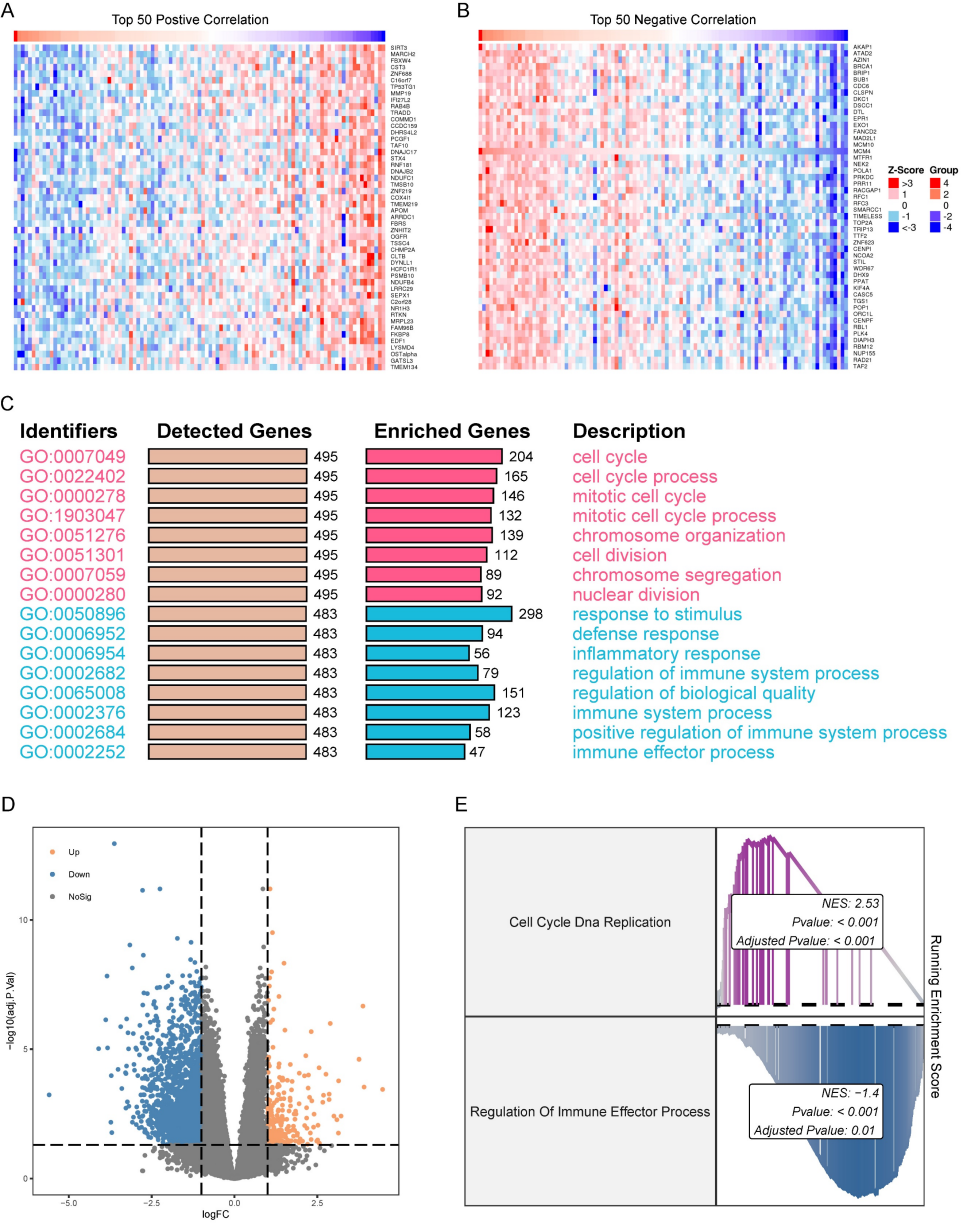
** Expression correlation and functional enrichment analysis in SKCM.** (A) Heatmap of the top 50 genes co-expressed with MCM4. Expression values of genes are normalized to z scores. (B) Heatmap of the top 50 genes negatively correlated with MCM4. (C) Gene Ontology (GO) terms associated with both positively and negatively correlated genes. Red indicates the functions involved in positively correlated genes. Blue indicates the functions involved in negatively correlated genes. (D) Volcano plot of the two MCM4-related subgroups. (E) GSEA results of Cell Cycle DNA Replication and Regulation of Immune Effector Process.

To identify genes co-expressed with MCM4 in the TCGA-SKCM cohort, we performed Spearman correlation analysis. Heatmaps were generated to display the top 50 genes positively and negatively correlated with MCM4 (Figure 5A, B). Next, we performed GO enrichment analysis on the top 500 co-expressed genes. The results revealed that genes positively correlated with MCM4 were predominantly involved in cell cycle-related pathways, while genes negatively correlated with MCM4 were enriched in immune-related processes (Figure 5C). Based on the median expression of MCM4, SKCM patients were divided into high-expression and low-expression groups. Differential analysis was performed using the limma package 30, and a threshold of |log2FC >= 1| and adjusted p-value < 0.05 was applied to identify 1867 differentially expressed genes (DEGs) (Figure 5D). The GSEA algorithm demonstrated significant upregulation of "Cell Cycle DNA Replication" and downregulation of "Regulation of Immune Effector Process" in the high MCM4 expression group (Figure 5E).

### TME analysis

Understanding the interactions between tumor cells and the immune system, as well as the evolving TME, is essential for advancing immunotherapy development 26, 31. Enrichment analysis revealed that MCM4 negatively regulates immune responses, prompting a deeper investigation into its impact on TME in SKCM.

**Figure 6:**
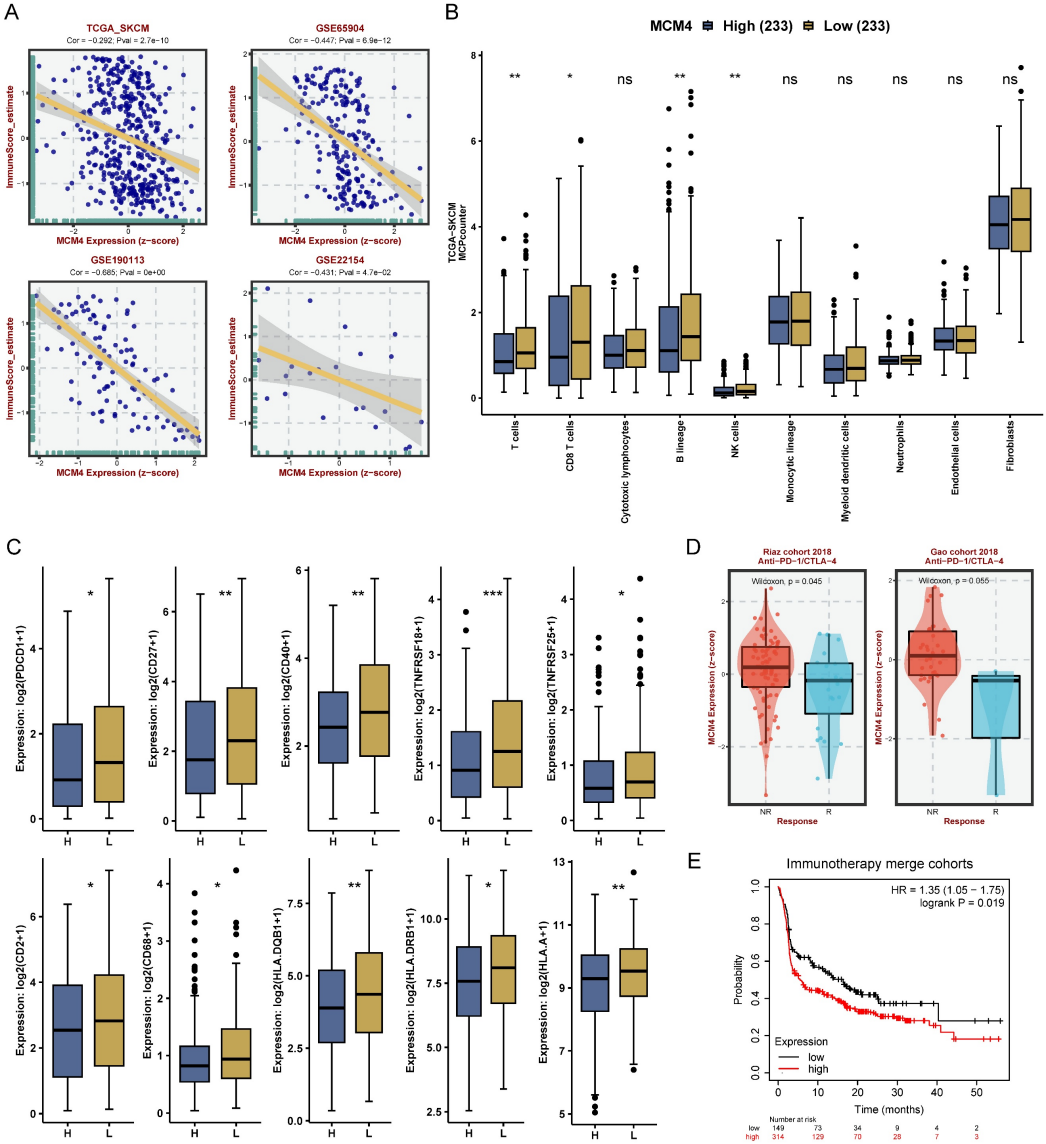
** MCM4 modulates immune infiltration and predicts immunotherapy response in SKCM.** (A) Spearman analysis between MCM4 and immune score in TCGA-SKCM and GEO datasets. (B) Tumor microenvironment pattern between the high- and low-MCM4 subgroups. (C) Differential expression of immune checkpoint molecules between the high- and low-MCM4 subgroups. (D) Expression levels of MCM4 in response (R) and non-response (NR) patients. (E) Survival analysis in the immunotherapy cohort suggesting potential associations of low MCM4 expression with improved patient outcomes. H: high MCM4 group, L: low MCM4 group. *p < 0.05, **p < 0.01, ***p < 0.001.

Our analysis revealed a consistent negative correlation between MCM4 expression and immune scores across the TCGA-SKCM dataset and three GEO cohorts (Figure 6A). Next, we calculated the TME components in the TCGA-SKCM cohort using the MCPcounter algorithm. Compared with the low-MCM4 group, the high-MCM4 group showed lower infiltration levels of T cells, CD8+ T cells, B lineage cells, and NK cells (Figure 6B). We also found that the high-MCM4 group had lower expression levels of immune checkpoint (Figure 6C). Furthermore, MCM4 expression was lower in patients who responded to immunotherapy (Figure 6D). KM curves suggested that patients with elevated MCM4 expression had shorter OS (Figure 6E). Our results indicate that MCM4 has the potential to serve as a novel biomarker for immunotherapy in SKCM.

### scRNA-seq and spatial transcriptome analysis

To gain a more detailed understanding of MCM4 expression in the SKCM microenvironment, we analyzed the scRNA-seq dataset GSE115978. We identified nine cell types: malignant cells, B cells, monocytes/macrophages, endothelial cells, fibroblasts, natural killer cells, proliferating T cells, exhausted CD8 T cells, and conventional CD4 T cells (Figure S2A). MCM4 expression was predominantly found in tumor cells and proliferating T cells (Figure S2B, C).

We further analyzed the Human Melanoma IF Stained (FFPE) dataset from the 10X Genomics database (https://www.10xgenomics.com/). Using deconvolution methods, we calculated the content and localization of all cell types in microregions (Figure S2D). Expression analysis and comparative assessment revealed that MCM4 was primarily expressed in malignant cells (Figure S2E, F). Correlation heatmap revealed that MCM4 expression was positively associated with tumor cells, while exhibiting a significant negative correlation with immune cells such as CD4+ T cells and CD8+ T cells (Figure S2G). Further analysis of spatial transcriptome data from the GSE179572 dataset corroborated these findings, aligning closely with results from the 10X cohort (Figure S2H-K). These findings further support the potential role of MCM4 as a biomarker for tumor progression and immune evasion in SKCM.

### Functional experiments of MCM4 knockdown in two SKCM cell lines

**Figure 7:**
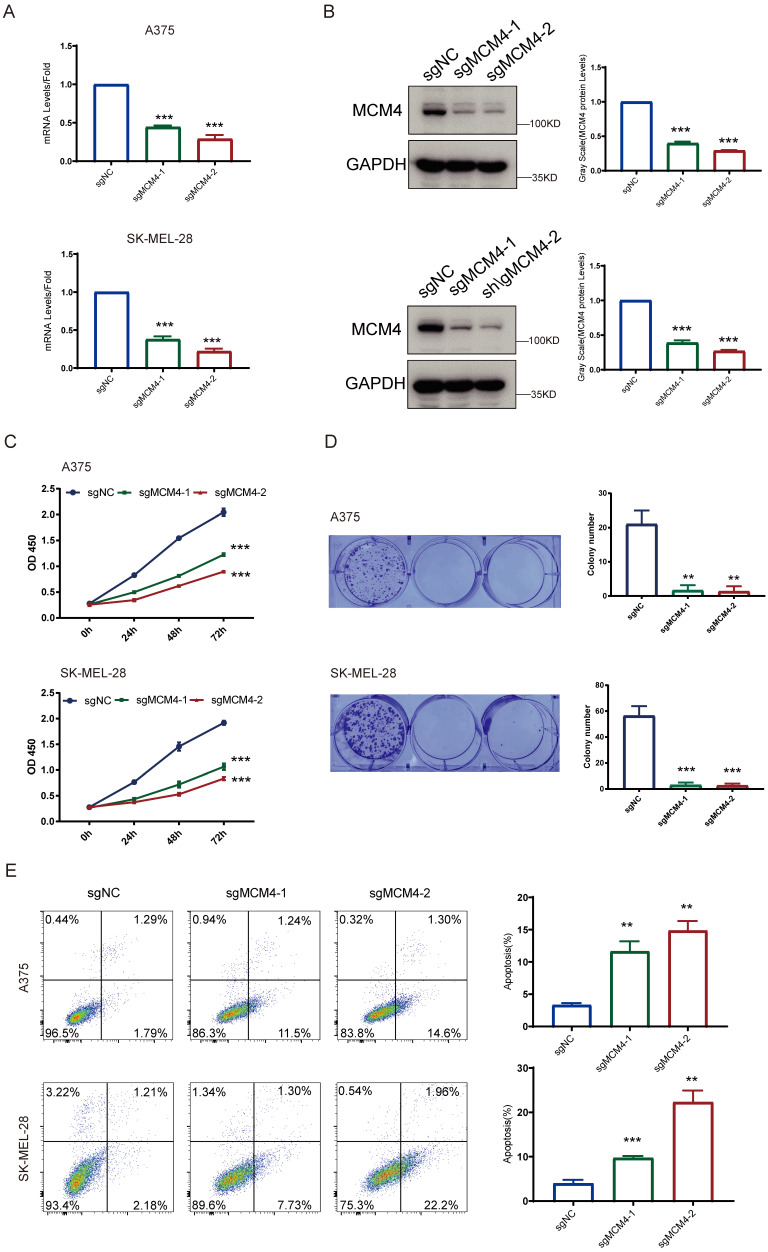
** MCM4 knockdown inhibits SKCM cell growth and induces apoptosis.** (A) The qPCR results of MCM4 mRNA levels in SKCM cells. (B) WB analysis of MCM4 protein expression. (C) CCK8 assay assessment of cell growth in SKCM cells. (D) Colony formation assay of SKCM cells with or without MCM4 knockdown. (E) Flow cytometry analysis of apoptosis in transfected SKCM cells.

To explore the functional role of MCM4 in SKCM, we performed knockdown experiments. The qPCR analysis confirmed that both sgRNAs significantly decreased MCM4 mRNA levels relative to controls, and WB assays validated effective suppression of MCM4 protein expression in both A375 and SK-MEL-28 cells (Figure 7A, B). The impact of MCM4 knockdown on cell proliferation was subsequently assessed using CCK-8 and colony formation assays. These assays revealed a significant decrease in both cell proliferation and survival rates in the MCM4 knockdown groups compared to the respective control groups (Figures 7C, D). Flow cytometry analysis further demonstrated a higher cell apoptosis in the MCM4 knockdown group (Figure 7E). These results indicate that MCM4 plays a key role in promoting SKCM cell proliferation.

### Prediction of anti-tumor drug

**Figure 8:**
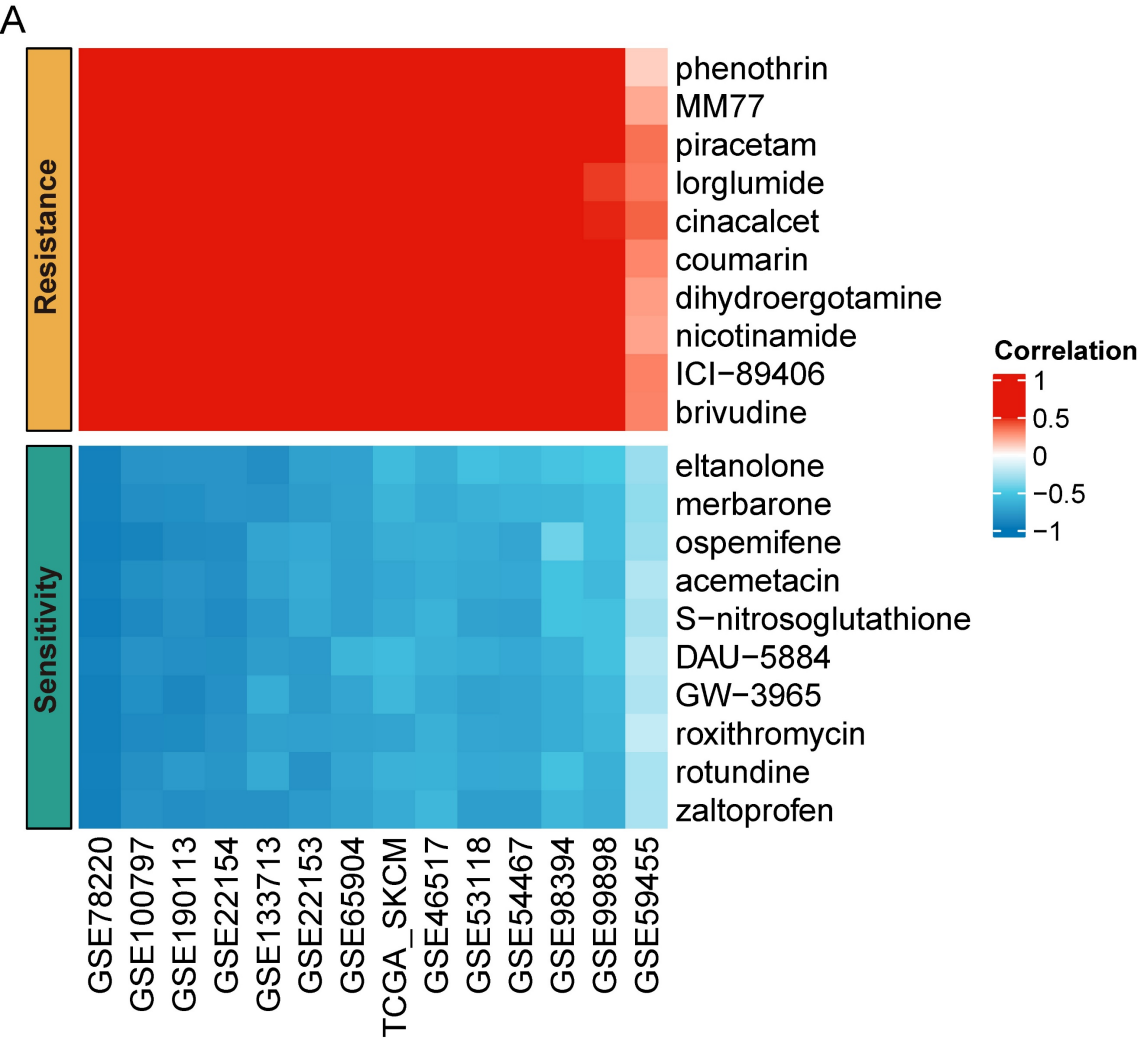
** Predictions of drug susceptibilities based on MCM4 expression across SKCM datasets.** (A) Spearman analysis indicating the correlation between MCM4 and sensitivity to potential drugs in SKCM.

Targeted therapy and chemoradiotherapy remain vital treatments for melanoma 1, 32. To enhance SKCM care, we performed a drug sensitivity prediction across multiple SKCM cohorts using pharmacogenomic data from the PRISM database. Our analysis revealed that MCM4 expression may serve as a biomarker for drug response. SKCM patients with high MCM4 were more sensitive to eltanolone, merbarone, ospemifene, acemetacin, S-nitrosoglutathione, DAU-5884, GW-3965, roxithromycin, rotundine, and zaltoprofen, while showing resistance to phenothrin, MM77, piracetam, lorglumide, cinacalcet, coumarin, dihydroergotamine, nicotinamide, ICI-89406, and brivudine (Figure 8A). The consistent association of these drugs with MCM4 expression across multiple independent cohorts provides valuable insights into potential drug responses for SKCM patients.

## Discussion

In clinical practice, the prognosis assessment of SKCM patients primarily relies on histopathology parameters such as age, skin color, and tumor stage. While these clinical parameters contribute to some extent in the clinical management of patients, they still pose challenges 33, 34. Due to the highly metastatic nature of melanoma, there is an urgent need for new biomarkers and drug targets to improve the accuracy of melanoma diagnosis and treatment 3, 35. Addressing this clinical demand, we comprehensively investigated the expression of MCM4 in tumor tissue and metastatic tissue using multiple SKCM datasets. Our research also establishes the foundation for MCM4 as a pan-cancer biomarker.

We observed that MCM4 expression was not significantly elevated in several cancer types, such as KIRC and PRAD. This may be attributed to three main reasons. First, different tumors are biologically distinct, with varying molecular pathways involved in their progression 36, 37. This tumor heterogeneity can lead to diverse expression patterns of MCM4, which may be regulated differently based on the tumor microenvironment, genetic mutations, or epigenetic factors. Second, while MCM4 is involved in DNA replication and cell cycle regulation, its role is likely context-dependent. In some tumors, MCM4 may contribute significantly to cell proliferation and genomic instability, leading to higher expression levels, whereas in others, alternative mechanisms might compensate for its loss or downregulation. Finally, immune cell infiltration in the TME may influence MCM4 expression, as tumors with higher immune cell infiltration often exhibit altered gene expression profiles due to the release of cytokines and other modulatory molecules by immune cells 38, 39. These factors suggest that the degree of immune cell infiltration may affect MCM4 expression, and further studies are warranted to explore the underlying mechanisms in different tumors.

We observed that MCM4 expression was not significantly elevated in several cancer types, such as KIRC and PRAD. This may be attributed to three main reasons. First, different tumors are biologically distinct, with varying molecular pathways involved in their progression 40, 41. This tumor heterogeneity can lead to diverse expression patterns of MCM4, which may be regulated differently based on the tumor microenvironment, genetic mutations, or epigenetic factors. Second, while MCM4 is involved in DNA replication and cell cycle regulation, its role is likely context-dependent. In some tumors, MCM4 may contribute significantly to cell proliferation and genomic instability, leading to higher expression levels, whereas in others, alternative mechanisms might compensate for its loss or downregulation. Finally, immune cell infiltration in the TME may influence MCM4 expression, as tumors with higher immune cell infiltration often exhibit altered gene expression profiles due to the release of cytokines and other modulatory molecules by immune cells 38, 39. These factors suggest that the degree of immune cell infiltration may affect MCM4 expression, and further studies are warranted to explore the underlying mechanisms in different tumors.

In genomic analysis, we have observed multiple gene site mutations in MCM4 across pan-cancer. Although the overall frequency is low, these changes at these loci may increase the risk of developing tumors. In fact, Yukio et al. identified the MCM4 G364R mutation in skin cancer cells, which affects the DNA helicase activity of the MCM complex 42. Additionally, the mutation MCM4 F345I has been found to be associated with breast cancer 43. This mutation affects the entry of MCM4 into the cell nucleus and its formation of complexes with MCM4, MCM6, and MCM7 44. These findings suggest a potential association between malignant transformation of normal cells and abnormal chromosome structure.

We employed bioinformatics analysis and inhibitor experiments to identify SRF as a potential TF for MCM4. In previous studies, SRF has been shown to be activated by RAC1 P29S, initiating transcriptional programs that promote malignant progression of melanoma. The use of SRF inhibitors has been found to help overcome resistance to BRAF inhibitors in melanoma 45. Our research supports SRF as a therapeutic target and provides further insights into its role in SKCM. Future work can investigate this proposed regulatory mechanism in more depth.

Through correlation analysis and enrichment analysis, we investigated the potential mechanisms of MCM4 in SKCM. Among the genes positively correlated with MCM4, we found significant enrichment of pathways related to cell cycle and chromosome organization. This finding aligns with the known functions of MCM4 and provides validation of the accuracy of the bioinformatics analysis. Surprisingly, we discovered a significant negative correlation between MCM4 and immune effector processes.

TME analysis revealed that MCM4 impacts the infiltration of immune cells (e.g., CD8+ T cells) into tumor tissues. This finding underscores the potential inhibitory effect of MCM4 in immune regulation. Consistent with this, previous studies on lung cancer and endometrial cancer have reported a negative correlation between MCM4 and inflammation and CD8+ T cells 46, 47. While immunotherapy has significantly improved the treatment outcomes for SKCM, increasing patient response rates remains a pressing challenge. On one hand, the heterogeneity of tumors and immune tolerance necessitate effective biomarkers to guide immune therapies for melanoma patients 48. On the other hand, the tumor microenvironment inhibits immune cell infiltration, impeding anti-tumor immune treatments 49. The inverse correlation between MCM4 expression and immunotherapy response points to its potential dual role as a therapeutic target and predictive marker. Future research will explore the fundamental pathways through which MCM4 modulates immune responses.

Our study enhances therapeutic strategies based on MCM4 expression. For instance, our results suggests that SKCM patients with low MCM4 levels may benefit from coumarin treatment, given its reported therapeutic potential in SKCM, including efficacy, low toxicity, and synergistic effects with cisplatin 50-52. Similarly, the drug GW-3965, which has shown sensitivity in patients with high MCM4 expression, demonstrated strong inhibition of tumor growth and metastasis in mice 53. However, targeting MCM4 for cancer therapy presents both promise and significant challenges. A key hurdle is the high structural conservation within the MCM2-7 complex, making the development of highly specific MCM4 inhibitors difficult. Off-target effects on other MCM subunits could disrupt essential cellular processes, leading to toxicity. One potential strategy to overcome this challenge is to focus on unique protein-protein interaction interfaces involving MCM4, rather than targeting the highly conserved ATPase domain. Disrupting these interactions might selectively impair MCM4 function without completely abolishing the activity of the entire complex. Another challenge lies in the potential for systemic toxicity due to the essential role of MCM4 in normal cell proliferation. Strategies such as tumor-targeted drug delivery systems, including antibody-drug conjugates or nanoparticle-based delivery, could minimize off-target effects and enhance therapeutic efficacy. Additionally, exploring combination therapies with DNA damage-inducing agents or checkpoint inhibitors could potentially synergize with partial MCM4 inhibition and improve treatment outcomes. While significant research is still needed, targeting MCM4 remains a promising avenue for developing novel cancer therapies.

It must be acknowledged that our study has certain limitations. Firstly, our findings are based on bioinformatics analysis of publicly available datasets, and they need to be validated in real-world cohorts using techniques such as western blot and immunohistochemistry. Secondly, the prognostic value of MCM4 in immunotherapy needs to be prospectively tested in larger patient populations. Lastly, our functional experiments were limited to cell lines, and further *in vivo* experiments are necessary.

In conclusion, our comprehensive analysis has established the clinical relevance of MCM4 in SKCM, providing novel insights into its oncogenic mechanisms and its role in immune regulation. Future studies are crucial to translate these findings into clinical practice by determining MCM4's utility as a biomarker in SKCM.

In conclusion, the comprehensive multi-omics analysis of MCM4 has established its clinical significance in SKCM and provided new insights into its oncogenic mechanisms and immune regulatory role. Further research is needed to determine the clinical utility of MCM4 as a biomarker and guide drug treatment in SKCM patients.

## Supplementary Material

Supplementary figures and table.
