# Supplementary material for: Comprehensive analysis reveals MCM4 as a biomarker for guiding therapies and immunomodulatory role in skin cutaneous melanoma
Source: J Cancer. 2025 Aug 11;16(12):3752–66. doi: 10.7150/jca.117471 (PMC12435266; doi:10.7150/jca.117471)
Supplement: Supplementary file 1 — Supplementary figures and table. [file jcav16p3752s1.pdf]

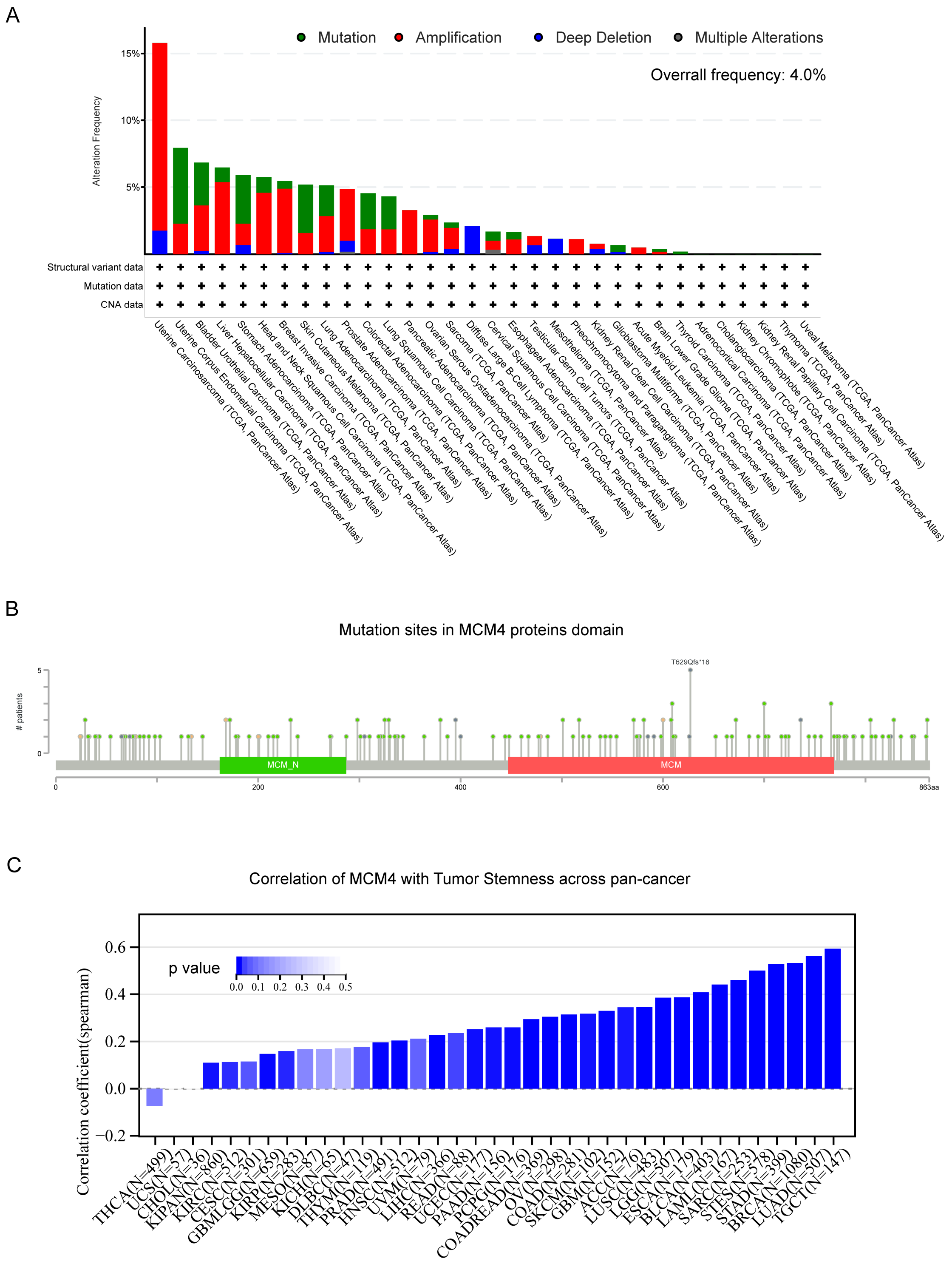

Figure S1. Genomic alteration profiles of MCM4 and its association with tumor stemness across various cancer types. (A) The genomic alteration frequencies and types of MCM4 in pan-cancer. (B) Relationship of mutation sites with domain in the MCM4 protein. (C) Correlation between MCM4 expression and tumor stemness across

pan-cancer. MCM-N: minichromosome maintenance complex (MCM) N-terminal domain (162 - 287). MCM: MCM domain (447 – 769).

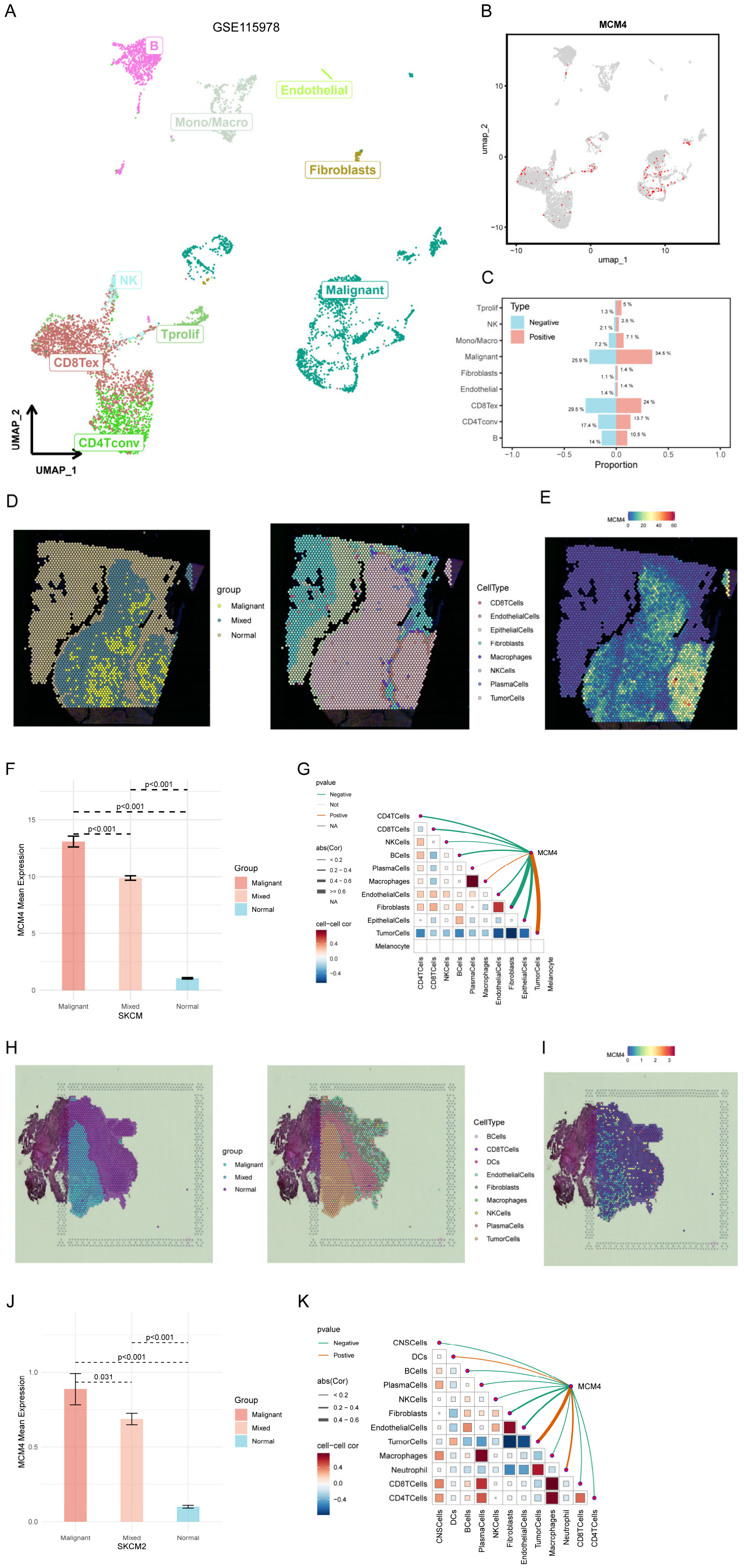

A
GSE115978
B
Mono/Macro
Endothelial
Fibroblasts
NK
Malignant
Tprolif
CD8Tex
CD4Tconv
UMAP_2
UMAP_1
B
MCM4
umap_2
umap_1
C
Type
Negative
Positive
Proportion
D
group
Malignant
Mixed
Normal
CellType
CD8TCells
EndothelialCells
EpithelialCells
Fibroblasts
Macrophages
NKCells
PlasmaCells
TumorCells
E
F
MCM4 Mean Expression
p<0.001
SKCM
Group
G
pvalue
abs(Cor)
cell−cell cor
H
I
J
SKCM2
0.031
K

Figure S2. scRNA-seq and spatial transcriptome analysis of MCM4 in SKCM. (A) Identification of various cell types in the GSE115978 dataset. (B) Visualization of MCM4 expression levels across different cell types. (C) The proportion of each cell type in the MCM4 expression positive group and negative group. (D) Localization of various cells after spatial transcriptome deconvolution in 10X cohort. (E) Expression levels of MCM4 across the spot. (F) Differential expression of MCM4 in malignant, mixed, and normal areas. (G) Spearman correlation between MCM4 and microenvironmental components. (H) Localization of various cells in GSE179572 dataset. (I) Expression levels of MCM4 across the spot. (J) Comparison of MCM4 in malignant, mixed, and normal areas. (K) Spearman correlation between MCM4 and microenvironmental components.

Supplement Table1. Sample information for the SKCM datasets

Variable	TCGA-SKCM	GSE46517	GSE98394
**Patients**	461	104	51
**Age**	58 (15, 90)	59 (28, 93)	61 (19, 88)
**Gender**			
Female	38%	34%	39%
Male	62%	66%	61%
